# A fusion-based multiomics classification approach for enhanced gene discovery in non-small cell lung cancer

**DOI:** 10.1093/bioadv/vbag131

**Published:** 2026-05-11

**Authors:** Kountay Dwivedi, Amirreza Mahbod, Rupert C Ecker, Klara Janjić

**Affiliations:** Center for Clinical Research, University Clinic of Dentistry, Medical University of Vienna, Vienna 1090, Austria; Research Center for Medical Image Analysis and Artificial Intelligence, Department of Medicine, Faculty of Medicine and Dentistry, Danube Private University, Krems an der Donau 3500, Austria; TissueGnostics GmbH, Vienna 1020, Austria; Center for Clinical Research, University Clinic of Dentistry, Medical University of Vienna, Vienna 1090, Austria

## Abstract

**Motivation:**

This study introduces a fusion-based multiomics approach to identifying non-small cell lung cancer (NSCLC)-relevant genes. We evaluated the NSCLC-subtype classification performance of various state-of-the-art machine learning models using single omics and fused multiomics approaches. The models were trained separately on individual omics datasets. Subsequently, a weighted-average-based decision-level fusion mechanism was employed to integrate the individual predictions of the trained models. Finally, the prediction performance across all the approaches was compared.

**Results:**

The decision-level fusion-based approach yielded a superior classification performance as compared to the performance achieved by models trained on individual omics datasets. Finally, a set of 47 NSCLC-relevant genes were identified. For the first time, *ABCF3*, *ACAP2*, *LSG1*, *TBCCD1*, *UCN2*, *WDR53*, *ZNF639* and *FYTTD1* appeared in the context of NSCLC. In conclusion, the integration of multiple omics types showed potential to deliver a more concise selection of NSCLC-relevant genes that could be clinically targeted in future.

**Availability and implementation:**

Data and source code are available on: https://github.com/kountaydwivedi/multiomics_fusion.git.

## 1 Introduction

Over the years, lung cancer has emerged as the most prevalent cancer worldwide, showing incremental growth in incidents and mortality. The Global Cancer Observatory (GLOBOCAN) 2025 survey estimates the number of new lung cancer incidences in the USA to be 226 650 (110 680 male; 115 970 female), out of which 124 730 (≈ 55%) are estimated mortality (64 190 male; 64 540 female) (Siegel *et al.* 2025). A 5-year survival rate of 10%–20% is generally observed, although timely clinical procedures such as chemotherapy and surgery have proven to be significantly effective in increasing this rate up to 60%–70% (Forde *et al.* 2025, Tang *et al.* 2025). Non-small cell lung cancer (NSCLC), being the most prominent lung cancer type, represents around 85% of the cases (Dwivedi *et al.* 2023). The advancements in molecular pathology further aided in the pathological-level subtype classification of NSCLC as adenocarcinoma (ADC) and squamous cell carcinoma (SCC) (Travis *et al.* 2015). This sub-categorization is based on the distinct and heterogeneous pathogenic characteristics portrayed by a characteristic gene expression profile of the tumor cells (Travis *et al.* 2015, Lipkova *et al.* 2022, Yang *et al.* 2022). It is, thus, imperative to identify the key genes accountable for the alterations leading to tumor development, subsequently deriving potential therapeutic targets (Travis *et al.* 2015, Girard *et al.* 2016, Chen and Dhahbi 2021, Dwivedi *et al.* 2023).

Throughout literature, several studies have contributed to shaping the landscape of NSCLC diagnosis, prediction and prognosis by investigating various omics-level data such as genomics and transcriptomics (Li *et al.* 2014, Girard *et al.* 2016, Qiu *et al.* 2017, Tian 2017, Chen and Dhahbi 2021, Dwivedi *et al.* 2023, 2024a, 2024b). The established works have followed a range of analytical approaches—from statistical methods such as correlation and Bayesian statistics (Girard *et al.* 2016, Khan *et al.* 2024) to computational frameworks such as genome-scale metabolic models (GEMS) (Huang *et al.* 2023, Bhowmick *et al.* 2024) and machine/deep learning-based artificial intelligence models (Dwivedi *et al.* 2023, 2024b).

While statistical techniques are computationally efficient and interpretable, thus bringing more transparency in the analysis, they often assume a linear or low-order relationship, missing complex, non-linear biological interactions (Rajula *et al.* 2020). Nevertheless, traditional statistical techniques are substantially useful when the sample space of the problem is significantly larger than the feature space (Rajula *et al.* 2020).

In contrast, GEMs are constraint-based flux analysis frameworks particularly employed to mathematically formulate cellular metabolism and physiological states in biological systems (Bernstein *et al.* 2021, Gonçalves *et al.* 2023, Chen *et al.* 2023). Although powerful computational tools to predict metabolic fluxes in living beings, GEMs confine to the limitation of inherent uncertainty in their predictions Bernstein *et al.* (2021). Further, the mapping of gene expression transcriptome information to reaction flux is a deep associative inference chain, where the gene expression is mapped to protein, which is further mapped to enzyme activity and then finally to flux. The missing omics information in the intermediary may lead to unreliable transcriptome-only-driven flux predictions (Kim and Lun 2014, Gonçalves *et al.* 2023).

While GEMs are typically considered knowledge-driven models, the data-driven machine/deep learning-based models are capable of handling large-scale omics datasets and learn complex, non-linear biological relationships. As a result, they have revolutionized the approach of developing omics and multiomics-based predictive models (Gonçalves *et al.* 2023, McLeish *et al.* 2024). Several notable research have demonstrated how machine/deep learning approaches can effectively be applied to omics-based data to identify NSCLC-relevant genes. For instance, multiple feature selection techniques such as minimum redundancy maximum relevance (mRMR) (Peng *et al.* 2005) and extreme gradient boosting (XGB) (Chen and Guestrin 2016) were employed to identify 17 NSCLC-relevant biomarkers. With a random forest classifier (Breiman 2001), the biomarkers exhibited an accuracy of 0.929 (Chen and Dhahbi 2021). Similarly, gene ranking methods including GeneRanks (Morrison *et al.* 2005) and RadViz (Hoffman *et al.* 1997) were employed to identify a set of eight biomarkers, subsequently resulting in an accuracy of 0.924 via support vector classifier (SVC) (Cortes and Vapnik 1995, Tian 2017). Furthermore, deep learning and SHapley Additive exPlanations (SHAP)-based interpretability techniques (Lundberg and Lee 2017a) were utilized for the discovery of 52 genes that assisted in achieving a prediction accuracy of 0.957 (Dwivedi *et al.* 2023).

The DNA copy number variation (CNV) acts as a key attribute in human genome development (Pfarr *et al.* 2016). The CNV information from the cancer genome may assist in identifying recurrent chromosomal alterations that, in turn, could aid in targeting cancer-related genes (Kim *et al.* 2013). Qiu *et al.* (2017) developed a custom algorithm that leveraged NSCLC-based CNV data to identify 33 biomarkers and achieve a subtype classification of 0.840. Further, Li *et al.* (2014) utilized mRMR to initially rank the NSCLC CNV probes, subsequently employed k-nearest neighbor (Fix and Hodges 1989) and incremental feature selection (Liu and Setiono 1998) algorithms to identify 266 probes. With the identified probes, they achieved a classification accuracy of 0.860 via leave-one-out cross-validation method. Dwivedi *et al.* (2024b) proposed a modified L_1_-regularization-based backpropagation algorithm, XL_1_R-Net, for NSCLC classification using CNV data. Using eXplainable AI (XAI) methods, the authors discovered a set of 20 NSCLC-relevant genes that predict NSCLC subtypes with an accuracy of 0.849.

Based on the aforementioned studies, it can be implied that individual omics data can sufficiently identify a set of NSCLC-relevant genes. Nonetheless, an intricate disease such as NSCLC exhibits various underlying interdependent biological characteristics that can be better interpreted by following a multiomics approach (Yang *et al.* 2022, Khadirnaikar *et al.* 2023, Steyaert *et al.* 2023). By fairly integrating a diverse set of omics data, a more sensitive and complementary set of genes can be identified that can be used subsequently to improve targeted therapy (Carrillo-Perez *et al.* 2022, Yang *et al.* 2022). This task can be accomplished by utilizing a decision-level fusion method such as hard/soft-voting scheme and weighted-average mechanism (Rastghalam and Pourghassem 2016, Qi *et al.* 2021, Carrillo-Perez *et al.* 2022). In these approaches, the outputs of isolated omics data are integrated or “fused” to attain a more stable and robust result. An established work demonstrated the potential of decision-level concept, where the authors utilized a weight-sum optimization method to fuse prediction probabilities obtained by analyzing transcriptomics, genomics, epigenomics as well as histological data of lung cancer. With the proposed method, they achieved a prediction accuracy of 0.955 (Carrillo-Perez *et al.* 2022).

Here, we hypothesized that, while omics data used in isolation can identify relevant genes, its efficacy is limited by the inability to adequately capture the inter/intra-biological dependencies. In light of this, the present work proposes the identification of key genes for NSCLC by integrating data from diverse omics types, particularly RNASeq-based gene expression transcriptomics and CNV-based genomics. To this end, we present a comprehensive benchmark study evaluating the classification performance of diverse, cutting-edge machine learning (ML) models trained on the aforementioned types of omics datasets, both individually and in a fused manner. We argue that the samples belonging to either of the NSCLC subtypes would be classified more accurately when the predictions derived from the two omics datasets are fused at the decision-level rather than used independently. This hypothesis would be falsified if models trained on individual omics datasets achieve equal or higher predictive accuracy than the fusion-based approach. Through this analysis, we further examine whether the fusion approach enhances the model’s ability to capture complex interconnected dependencies among features. Consequently, the most influential features contributing toward the classification could, therefore, be discovered and investigated for their clinical relevance in the diagnosis and prognosis of NSCLC.

**Figure 1:**
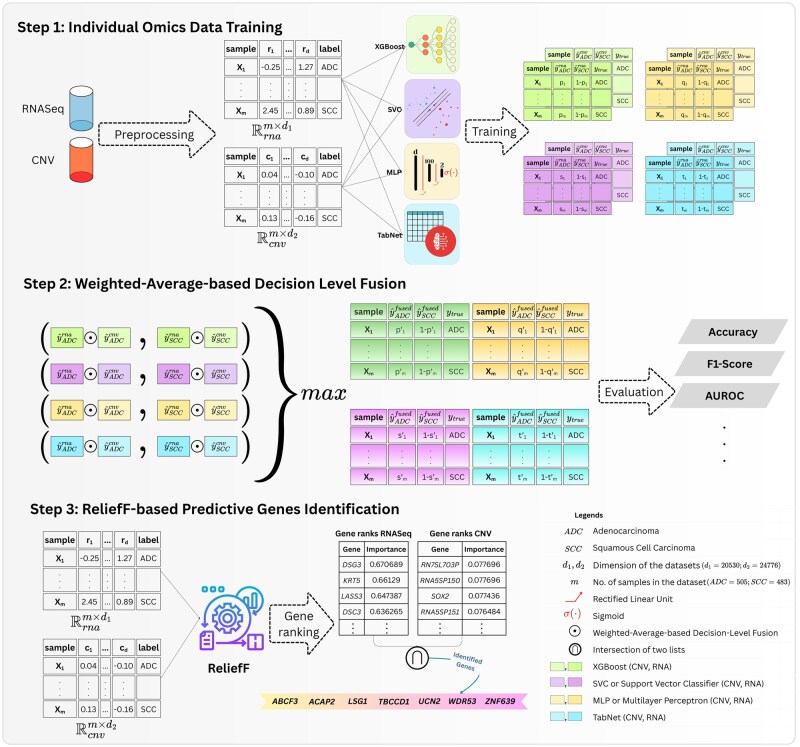
Methodological diagram. The experiment was conducted in three steps. The first step involved preprocessing of individual omics datasets. Thereafter, the preprocessed datasets were provided as inputs to individual machine learning models for training. At this stage, the predictive probabilities of each model were recorded. In the second step, the recorded predictive probabilities obtained from the first step were *fused* using the *weighted-average-based decision level fusion* concept. Subsequently, the evaluation of fusion-based approach was conducted and the results were compared with the outputs obtained from the individual models in the first step. Finally, the third step employed ReliefF method to compute the importance of gene set and ranked the genes according to their importance scores. Thereafter, the most important genes from the individual lists were intersected to identify the final list of NSCLC-relevant predictive genes.

The aim of this study was to discover a concise set of NSCLC-relevant genes using a multiomics approach. For this purpose, we evaluated the classification performance of diverse ML models, including XGB, SVC (Cortes and Vapnik 1995), multilayer perceptron (MLP) (Haykin 1994) and a transformer-based deep learning model TabNet (Arik and Pfister 2021) on publicly available ADC and SCC cohorts generated by The Cancer Genome Atlas (TCGA) program, comparing the performance based on individual as well as fused omics datasets. We also validated our approach on an independent cohort generated by Clinical Proteomic Tumor Analysis Consortium (CPTAC). Finally, the fused omics dataset is leveraged to identify a small set of NSCLC-relevant genes found most crucial in lung cancer classification. A descriptive representation of the methodological workflow followed in the experimentation is provided in Fig. 1.

## 2 Methods

### 2.1 Dataset and preprocessing

**Figure 2:**
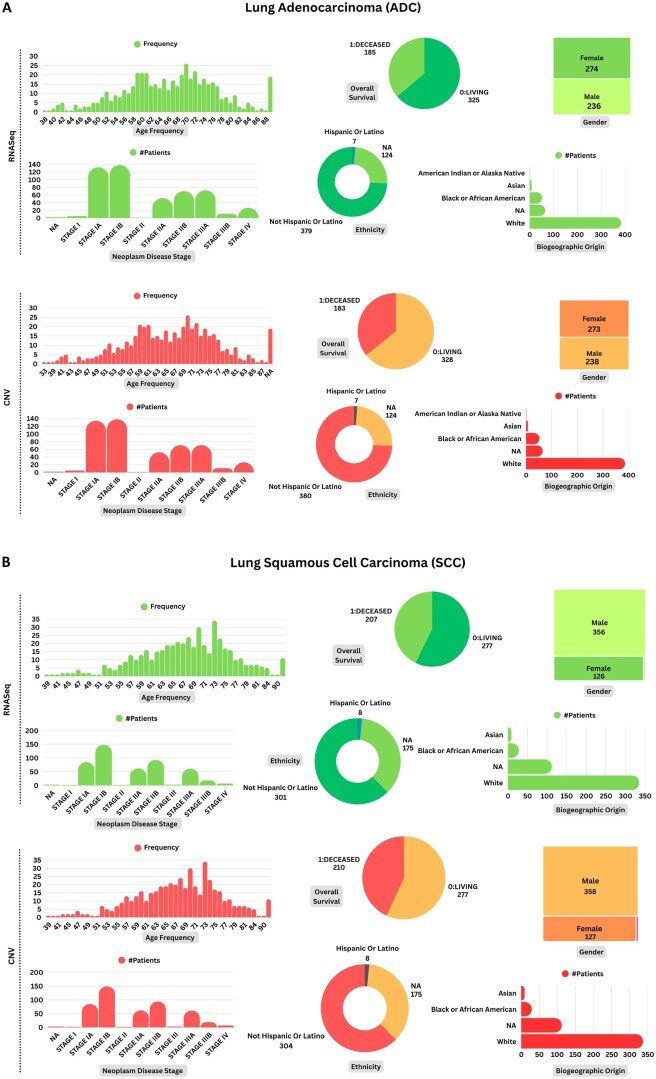
The demographic details of adenocarcinoma and squamous cell carcinoma patients falling under individual omics types. In the transcriptomics dataset, the demographic data were available for 510 out of 576 adenocarcinoma (ADC; A) patients and 484 out of 553 squamous cell carcinoma (SCC; B) patients. Similarly, in the genomics dataset, the demographic information was present for 511 out of 516 ADC patients and 487 out of 501 SCC patients. A complete summary of demographic and clinical characteristics is provided in Table 1, available as supplementary data at *Bioinformatics Advances* online.

The experimentation was performed on publicly available datasets generated by the TCGA (https://portal.gdc.cancer.gov/) program. The RNASeq and the CNV datasets were downloaded from the UCSC Xena (Goldman *et al.* 2020) repository on 27 December 2024. The number of samples per omics type for ADC and SCC is mentioned in Table 1, while the demographic details of the cohorts are summarized in Fig. 2 and Table 1, available as supplementary data at *Bioinformatics Advances* online.

**Table 1:** The number of samples and genes per omics type.^a^

TCGA dataset (training)	ADC	SCC	Genes
RNASeq gene expression	576	553	20 530
Copy number variation	516	501	24 776
*Common samples*	*505*	*483*	*–*
**CPTAC dataset (validation)**			
RNASeq gene expression	231	110	40 796
Copy number variation	232	110	38 275
*Common samples*	*227*	*110*	–

a An effective execution of the decision-level fusion mechanism necessitates the selection of samples with both types of omics data in the non-small cell lung cancer subtypes lung adenocarcinoma (ADC) and squamous cell carcinoma (SCC). We selected the common samples (represented in italics) from each subtype for the training as well as the independent validation datasets.

### 2.2 Omics data generation process

The UCSC Xena repository provided the process of generating various omics-level data. The RNASeq-based mRNA analysis performed to compute the expression level of individual genes in each sample under study was initiated by obtaining raw read counts aligned against the GRCh38 reference genome using the STAR software (Dobin *et al.* 2013) via HiSeq Illumina sequencers. Subsequently, the augmentation of read counts was performed using various transformation methods such as transcripts per million (TPM), fragments per kilobase of transcript per million mapped reads (FPKM) and upper quartile normalized FPKM (FPKM-UQ). The process was concluded by annotating the augmented read values with the gene symbols and gene biotype (Dobin *et al.* 2013), thereafter, computing the $log_{2}(\mathrm{count}+1)$ normalization of the read counts.

The CNV was estimated at the gene level by employing the GISTIC2 methodology (Mermel *et al.* 2011). Initially, the raw copy number values were generated using the Affymetrix single nucleotide polymorphism (SNP) 6.0 platform at the Broad Institute as part of the TCGA project. Subsequently, the obtained values were $log_{2}x-\mathrm{normalized}$ and segmented using the circular binary segmentation (CBS) (Olshen *et al.* 2004) algorithm. These segmented copy number values were conclusively mapped to the associated genes, resulting in gene-level copy number estimates. The segment-to-gene mapping was performed using the UCSC Xena HUGO probeMap (Mermel *et al.* 2011).

### 2.3 Validation cohort

While we performed our analysis based on derived TCGA-based datasets, we also validated the experimental results and findings on an independent cohort to measure the robustness across unseen data. Here, we utilized NSCLC ADC and SCC datasets generated by CPTAC (https://proteomics.cancer.gov/programs/cptac) program for validation. Both RNASeq and CNV datasets were downloaded from cBio Cancer Genomics Portal (cBioPortal) (De Bruijn *et al.* 2023) on November 2025. The details of the datasets are provided in Table 1.

### 2.4 Preprocessing

To implement an effective decision-level fusion mechanism, it was necessary to use the samples that contain both omics data. Accordingly, each omics dataset was initially examined to identify common samples across modalities. For this purpose, we utilized the TCGA sample ID barcodes provided in the dataset. The TCGA provides a unique barcode per sample that comprises multiple identifiers, as shown below:


$$\underset{\text{Project}}{\underset{︸}{\mathrm{TCGA}}}-\underset{\text{TSS}}{\underset{︸}{XX}}-\underset{\text{Participant}}{\underset{︸}{\mathrm{XXXX}}}-\underset{\text{Sample}}{\underset{︸}{XX}}$$

where TSS:Tissue Source Site (https://docs.gdc.cancer.gov/Encyclopedia/pages/TCGA_Barcode/). Using these barcodes, we identified the overlapping (or common) samples between RNASeq and CNV omics dataset within each NSCLC subtype. Specifically, we extracted a final set of 505 overlapping ADC samples and 483 overlapping SCC samples (Table 1). Evidently, the class imbalance was found almost negligible; hence, we did not implement any corrective measures such as over or under-sampling. Subsequently, when the datasets were investigated for missing values, no missing data were found. Finally, each dataset was normalized sample-wise using z-score normalization to scale its mean and standard deviation to zero and one, respectively.

For CPTAC validation datasets, we carried out the previously described preprocessing steps, except data normalization to prevent data leakage. Like TCGA, the CPTAC program assigns a unique ID to each sample that was leveraged to identify overlapping samples within each NSCLC subtype. In addition, we used the online SynGo (https://www.syngoportal.org/convert) (Koopmans *et al.* 2019) portal to convert Entrez Gene IDs to HGNC Gene IDs as the CPTAC datasets include the Entrez Gene IDs as feature identifiers.

### 2.5 Model training, validation, and decision-level fusion

For the purpose of training and validation, the state-of-the-art ML models were employed, including XGB, SVC, MLP and the transformer-based TabNet model. All the models were utilized with their default hyperparameter settings (Table 2).

**Table 2:** Parameter settings of models.^a^

Model	Hyperparameters
**XGB**	*booster=gbtree; eta=0.3;*
	*gamma=0; lambda=1; alpha=0;*
	*tree_method=auto*.
**SVC**	*C=1.0; kernel=rbf;*
	*degree=3; gamma=scale; probability=True*.
**MLP**	*hidden_layer_sizes=100;*
	*activation=relu; solver=adam;*
	*batch_size=auto; learning_rate=constant;*
	*learning_rate_init=0.001; alpha=0.0001;*
	*max_iter=200; early_stopping=False*.
**TabNet**	*eval_metric=[auc, accuracy];*
	*batch_size=512; patience=0;*
	*n_d=8; n_a=8; n_steps=3; gamma=1.3;*
	*optimizer_fn=adam;*
	*optimizer_params=2e-2; epsilon=1e-15*.

a The list of various hyperparameter values of individual models employed in the experiment. All the hyperparameters were kept to their default value.

Initially, each model was trained separately on individual omics datasets. To ensure a robust model selection, the training was performed using stratified *k*-fold cross validation, with k=5. To prevent data leakage, the optimized tool *StratifiedKFold* from *scikit-learn* library was employed.

It is to be noted that to mitigate the inherent stochastic nature of the computing system and provide more generalized results, the model training, validation and fusion mechanism was performed five times—each time using a different random seed value.

### 2.6 Weighted-average-based decision-level fusion mechanism

To ensure the accurate integration of the predictive probabilities computed from individual models, the same samples were maintained in the train and validation splits across all the omics datasets. Formally, for NSCLC subtype prediction—a binary classification problem—consider a set of training samples (in train split) $X_{t}$ and validation samples (in validation split) $X_{v}$, each with an associated class label $y_{t},y_{v}\in \{0,1\}$. Assume F(·) as the predictive model and i∈{RNASeq,CNV}. Then, for each $t\in X_{t}$ and $v\in X_{v}$:


 accR:=accuracy(Fi(t));i=RNASeq accC:=accuracy(Fi(t));i=CNV pRy=0,pRy=1:=prob(Fi(v));i=RNASeq pCy=0,pCy=1:=prob(Fi(v));i=CNV

Subsequently, each computed predictive probability is synergized with the associated weights $w_{R}$ and $w_{C}$, followed by the computation of decision-level fused prediction $\tilde{y}$:


(1) $$\alpha =(|e^{acc_{C}}-e^{acc_{R}}|)\cdot k$$

(2) $$w_{R}=|e^{\alpha \cdot acc_{R}}|/(|e^{\alpha \cdot acc_{R}}|+|e^{\alpha \cdot acc_{C}}|)$$

(3) $$w_{C}=|e^{\alpha \cdot acc_{C}}|/(|e^{\alpha \cdot acc_{R}}|+|e^{\alpha \cdot acc_{C}}|)$$

(4) $$pF_{y=0}=(w_{C}\cdot pC_{y=0})+(w_{R}\cdot pR_{y=0})$$

(5) $$pF_{y=1}=(w_{C}\cdot pC_{y=1})+(w_{R}\cdot pR_{y=1})$$

(6) $$\tilde{y}=\max(pF_{y=0},pF_{y=1})$$

where *k* is the scaling factor. The value of *k* was optimized empirically over the integer values ≥2 on a linear scale (see Fig. 1, available as supplementary data at *Bioinformatics Advances* online). It was observed that the lower values of *k* resulted in relatively lower performance of the models. A gradual increase in the classification performance was observed on increasing the value, eventually flattening the curve above the threshold value of 10. The final value of *k* was thus selected as 10 for the rest of the experimentation.

### 2.7 ReliefF-based predictive genes identification

In order to identify a concise set of genes most crucial in NSCLC subtype prediction, we used the ReliefF (Kononenko *et al.* 1997) method, implemented in the *scikit-rebate* (Urbanowicz *et al.* 2018) library. As highlighted by Urbanowicz *et al.* (2018), Relief-based algorithms are robust feature selection algorithms highly capable of learning complex feature dependencies, such as gene-gene interactions, in predictive modeling tasks. The original Relief method estimates the importance of an attribute based on how distinct its value is among the samples near to each other (Kononenko 1994). Assume there is a sample *X* belonging to a class Y∈{0,1} with a set of attributes with cardinality *M*. Relief initially searches two nearest neighbors of *X—Hit*, belonging to the same class as *X* (called nearest hit) and *Miss*, belonging to the other remaining class (called nearest miss). Assume *n* to be a subset of randomly selected samples, the weights initialization takes places as presented in Algorithm 1:

Algorithm 1 1: set all weights W[M]:=0.0

2: **for all** *n* **do** 

3:   randomly select an instance *X*

4:   find nearest hit *Hit*

5:   find nearest miss *Miss*

6:   **for**  A←[1⋯M]  **do** 

7:    $W[A]:=W[A]-\frac{\mathrm{diff}(A,X,\mathrm{Hit})}{n}+\frac{\mathrm{diff}(A,X,\mathrm{Miss})}{n}$

8:   **end for** 

9: **end for** 

Unlike the original Relief method, the ReliefF searches for k−nearest hits/misses instead of just one, thereby increasing the reliability of the resultant weights-approximation (Kononenko *et al.* 1997).

**Figure 3:**
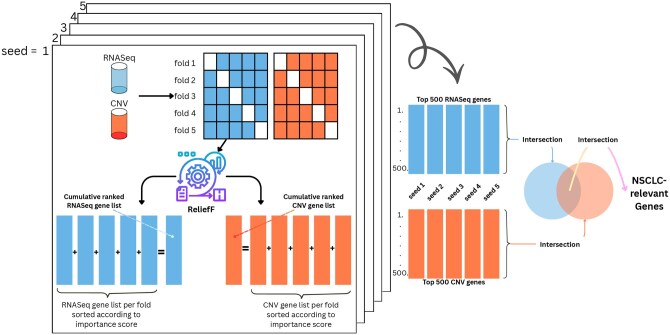
ReliefF-based predictive genes identification. Initially, we performed a stratified five-fold cross-validation-based feature selection on individual omics datasets. Feature selection and model training were conducted exclusively on the train split of each fold, while evaluation was performed on the corresponding validation split. After completing all five folds, a set of five gene-importance lists for each omics type was obtained, i.e. a total of 10 lists—five for transcriptomics and five for genomics type. For each omics type, the corresponding lists were then sorted by importance scores and aggregated to produce a final ranked list of genes, with cumulative importance scores computed across the folds. This process was repeated five times, each time with a different random seed value, producing 10 ranked gene lists in total—five for the transcriptomics data and five for the genomics data. From each ranked list, we selected the top 500 genes. These five lists within each omics type were intersected to obtain omics-discriminative stable gene sets. Finally, we intersected the resulting transcriptomics-discriminative and genomics-discriminative sets to identify the genes that were consistent across both omics types.

The entire predictive gene-selection process is illustrated in Fig. 3. We performed a stratified five-fold cross-validation-based feature selection with ReliefF (using *scikit-learn* and *skrebate* libraries, respectively) for better and robust predictive genes while keeping train and test splits separately. Specifically, we computed feature selection separately for each omics type using the *scikit-learn StratifiedKFold* tool with *k *= 5. For each fold, the data were split into train split (80%) and test split (20%) in a stratified manner. Feature selection and model training were conducted exclusively on the train split of each fold, while validation was performed on the corresponding test split. After completing all five folds, we obtained five gene-importance lists for each omics type, i.e. a total of 10 lists—five for transcriptomics and five for genomics type. For each omics type, the corresponding lists were then sorted by importance scores of genes and aggregated to produce a final ranked list of genes, with cumulative importance scores computed across the folds.

Furthermore, to evaluate the stability of ReliefF, we repeated the aforementioned feature-selection process five times, each time with a different random seed value. This resulted in 10 ranked gene lists-five for the transcriptomics data and five for the genomics data. From each ranked list, we selected the top 500 genes. We then intersected the five lists within each omics type to obtain omics-discriminative stable gene sets. Finally, we intersected the resulting transcriptomics-discriminative and genomics-discriminative sets to identify the genes that were consistent across both omics types.

### 2.8 Computation of predictive performance of the identified genes

To assess the predictive performance of the identified set of genes, we utilized the independent CPTAC cohort (validation dataset). The model used for evaluation was the best performing model among XGB, SVC, MLP and TabNet when trained and validated on the TCGA dataset. Initially, we retained only the identified set of genes and discarded the rest from both transcriptomics and genomics datasets. Subsequently, we evaluated the classification performance of the identified genes on individual omics datasets. We also validated our proposed fusion-based approach to investigate its effectiveness on unseen data. We performed stratified *k*-fold cross-validation with k=5 (using *StratifiedKFold* method from *scikit-learn* library) and the metrics used for evaluation were accuracy and area under the ROC curve (AUROC) scores. For a robust validation, we iterated the experiment five times, each with a different random seed value and the metrics were computed by taking an average over all the five iterations.

#### 2.8.1 SHAPley analysis of identified genes

We further conducted a SHAP (Lundberg and Lee 2017a) analysis to quantify and visualize the contributions of the identified set of genes toward the classification. SHAP is an XAI tools that is based on *cooperative game theory* concept proposed by Lloyd Shapley in 1953 (Shapley 1953). The idea, in context of ML, elucidates how much a feature contribute toward individual prediction.

For the SHAP analysis, we utilized the CPTAC validation cohort. The XGB model was used for training and validation, as it exhibited superior performance compared to the other employed models. Initially, we retained only the identified genes in the individual omics datasets in the CPTAC cohort. Next, we employed a train and hold-out set concept for this experiment, wherein the individual omics datasets were split into two parts—train and hold-out subsets. For this purpose, we used the *train_test_split* method provided in the *scikit-learn* library, splitting the datasets into 80% train and 20% hold-out subsets. Care was taken to split the datasets in a stratified manner using the *stratify* hyperparameter of *train_test_split* method. The model training and the SHAP analysis was performed on the train and held-out subsets, respectively. For robust analysis, the experiment was iterated for five times, each with a different random seed value. Subsequently, the mean absolute SHAP value of each gene (obtained across all five iterations) was reported as the final SHAP value.

### 2.9 Functional enrichment analysis of the identified genes

Having discovered the crucial subset of NSCLC-relevant genes, we proceeded to investigate their biological relevance. Initially, we explored the functional enrichment of the discovered set of genes. This analysis was performed in terms of gene ontology (GO) and biological pathways by utilizing the online available WEB-based GEne SeT AnaLysis Toolkit (WebGestalt) (Zhang *et al.* 2005). The databases utilized to analyze the functional pathways were Protein ANalysis THrough Evolutionary Relationships (PANTHER) (Mi and Thomas 2009), Kyoto Encyclopedia of Genes and Genomes (KEGG) (Kanehisa and Goto 2000) and Reactome (Fabregat *et al.* 2017).

### 2.10 Overall survival-based prognostic efficacy of the identified genes

Post-investigating the functional and biological enrichment of the discovered genes, we investigated their survival prediction performance. The evaluation of the prognostic efficacy of the discovered set of genes was carried out by utilizing the online Kaplan–Meier (KM) Plotter tool (Györffy *et al.* 2010), which provides transcriptome profiles of large sample sets from multiple cancer cohorts and performs real-time survival analysis by employing Cox regression and KM plots. For this analysis, the lung cancer cohort from the KM Plotter database, comprising 1411 patients, was utilized. The discovered set of 47 genes was provided as an input and the overall survival was computed using the default parameters set by the platform. The survival time was recorded in months.

### 2.11 Implementation details

The ML models, including XGB, SVC and MLP, were employed using the *scikit-learn v1.4.2* library. To implement the transformer-based TabNet model, the *pytorch-tabnet v4.1.0* library was utilized. The hyperparameter settings of each model was set to default, as mentioned in Table 2. Each model was initially trained on individual omics dataset using stratified five-fold cross validation. This partitioned the entire dataset into five folds, i.e. within each fold, the samples were divided into 80% train split and 20% validation split, while ensuring balanced class ratio of both ADC and SCC samples. A similar trend was followed in case of independent CPTAC datasets for validation purpose.

For ReliefF implementation, we utilized the *scikit-rebate v0.6* library. The ReliefF method implicitly utilizes *k-nearest neighbors* algorithm for feature ranking. The hyperparameter *k*, which denotes the number of nearest neighbors to consider, was set to its default value 100.

For a robust analysis, within each experiment, we performed a stratified five-fold cross-validation, efficiently splitting the entire dataset into 80% train subset and 20% validation subset. With this setting, each instance in the dataset gets a chance to be validated upon. Furthermore, we ran the entire experimentation for five iterations, each time with a different random seed value. This ensured that the model weights get initialized distinctly as per the inherent stochasticity of the hardware.

The entire experimentation was performed using *Python v3.12.3* and *Pytorch v2.5.1* on a Windows 11 Pro platform with 64GB RWM and 12th Gen Intel Core i9-12900 processor, clocked at 2.4 GHz. The system comprised a dedicated 8GB NVIDIA GeForce RTX 3070 GPU with CUDA *v12.4*. The processed training and validation datasets along with the implementation codes are provided in the GitHub repository: https://github.com/kountaydwivedi/multiomics_fusion.git.

## 3 Results

### 3.1 Prediction performance comparison of individual models with decision-level fusion approach

To accomplish an accurate decision-level fusion mechanism, the ML models were first trained on individual omics datasets. This approach facilitated a comprehensive comparison of the predictive performance of individual omics data with the fusion-based approach. The performance was evaluated using a confusion matrix and the AUROC score. The confusion matrix provided a detailed segregation of the predicted labels compared to the true labels. The matrix comprises four cells:

- **True positives (TP):** The correctly predicted positive samples.
- **False positives (FP):** The incorrectly predicted positive samples; also known as Type-I error.
- **True negatives (TN):** The correctly predicted negative samples.
- **False negatives (FN):** The incorrectly predicted negative samples; also known as Type-II error.

With the help of the confusion matrix, various classification metrics were derived, including accuracy, precision (positive predicted values or PPV), recall, specificity, F1-score and negative predicted values (NPV).

The receiver operator characteristic (ROC) curve in the AUROC plots the true positive rate (TPR) against the false positive rate (FPR) at various classification thresholds, essentially segregating the “signal” from the “noise.” The AUROC summarizes the ROC curve by providing the measure of the ability of the model to distinguish between the classes (McClish 1989). Recall that the models were trained across multiple random seeds to capture the inherent randomness of the system. Therefore, the final confusion matrix and the AUROC curve scores were computed as the average of all the confusion matrices and AUROC curve scores obtained across different random seed iterations.

The comparative analysis of the aforementioned metrics of different models employed on individual datasets and that of the fusion-based approach revealed that the fusion-based approach outperformed the models trained on the individual datasets (Table 3). We further employed Mann-Whitney U test (Mann and Whitney 1947) to statistically compare the performance of the models trained on individual omics datasets and their fusion-based counterparts. We report the resultant *P*-value and the corresponding effect size in Table 3, available as supplementary data at *Bioinformatics Advances* online. The *P*-value indicates if the observed difference in the performance between two models being compared is statistically significant; a smaller *P*-value (generally p≤0.05) is considered significant. On the contrary, the effect size quantifies the magnitude of the observed difference—a smaller effect size indicates that the performance may be practically limited, even if the difference is statistical (Sullivan and Feinn 2012).

**Table 3:** Prediction performance of each individual omics dataset alongside fusion-based approach (mean ± standard deviation).^a^

Model/approach	Accuracy	AUROC	Precision (PPV)	Recall (sensitivity)	Specificity	F1-score	NPV
XGB-RNA	0.954${}_{\pm 0.001}$	**0.991** ${}_{\pm 0.000}$	0.942${}_{\pm 0.002}$	0.970${}_{\pm 0.001}$	0.938${}_{\pm 0.002}$	0.956${}_{\pm 0.001}$	0.968${}_{\pm 0.001}$
XGB-CNV	0.892 ${}_{\pm 0.005}$	0.953${}_{\pm 0.001}$	0.891${}_{\pm 0.003}$	0.899${}_{\pm 0.008}$	0.885${}_{\pm 0.003}$	0.895${}_{\pm 0.005}$	0.894${}_{\pm 0.008}$
**XGB-fusion**	**0.958** ${}_{\pm 0.001}$	0.982${}_{\pm 0.001}$	**0.947** ${}_{\pm 0.002}$	**0.972** ${}_{\pm 0.003}$	**0.943** ${}_{\pm 0.003}$	**0.959** ${}_{\pm 0.001}$	**0.970** ${}_{\pm 0.003}$
SVC-RNA	0.951${}_{\pm 0.000}$	**0.987** ${}_{\pm 0.000}$	0.928${}_{\pm 0.001}$	**0.980** ${}_{\pm 0.000}$	0.921${}_{\pm 0.001}$	**0.954** ${}_{\pm 0.000}$	**0.977** ${}_{\pm 0.000}$
SVC-CNV	0.920${}_{\pm 0.002}$	0.960${}_{\pm 0.000}$	0.900${}_{\pm 0.003}$	0.944${}_{\pm 0.001}$	0.894${}_{\pm 0.003}$	0.924${}_{\pm 0.001}$	0.939${}_{\pm 0.001}$
**SVC-fusion**	**0.952** ${}_{\pm 0.000}$	0.980${}_{\pm 0.000}$	**0.938** ${}_{\pm 0.001}$	0.971${}_{\pm 0.001}$	**0.932** ${}_{\pm 0.002}$	**0.954** ${}_{\pm 0.000}$	0.969${}_{\pm 0.001}$
MLP-RNA	0.800${}_{\pm 0.069}$	0.900${}_{\pm 0.060}$	0.786${}_{\pm 0.125}$	0.893${}_{\pm 0.117}$	0.703${}_{\pm 0.230}$	0.822${}_{\pm 0.042}$	0.892${}_{\pm 0.095}$
MLP-CNV	0.904${}_{\pm 0.004}$	0.958${}_{\pm 0.003}$	0.907${}_{\pm 0.004}$	0.906${}_{\pm 0.008}$	0.902${}_{\pm 0.005}$	0.907${}_{\pm 0.004}$	0.902${}_{\pm 0.007}$
**MLP-fusion**	**0.919** ${}_{\pm 0.011}$	**0.968** ${}_{\pm 0.004}$	**0.917** ${}_{\pm 0.006}$	**0.926** ${}_{\pm 0.019}$	**0.912** ${}_{\pm 0.006}$	**0.921** ${}_{\pm 0.012}$	**0.922** ${}_{\pm 0.019}$
TabNet-RNA	0.698${}_{\pm 0.038}$	0.782${}_{\pm 0.046}$	0.733${}_{\pm 0.065}$	0.655${}_{\pm 0.048}$	0.743${}_{\pm 0.084}$	0.690${}_{\pm 0.032}$	0.673${}_{\pm 0.030}$
TabNet-CNV	0.831${}_{\pm 0.012}$	**0.926** ${}_{\pm 0.005}$	0.891${}_{\pm 0.015}$	0.764${}_{\pm 0.038}$	0.902${}_{\pm 0.020}$	0.823${}_{\pm 0.016}$	0.786${}_{\pm 0.024}$
**TabNet-fusion**	**0.848** ${}_{\pm 0.010}$	0.925${}_{\pm 0.006}$	**0.903** ${}_{\pm 0.017}$	**0.788** ${}_{\pm 0.032}$	**0.911** ${}_{\pm 0.020}$	**0.841** ${}_{\pm 0.014}$	**0.805** ${}_{\pm 0.021}$

a A detailed description including the confidence intervals is provided in Table 2, available as supplementary data at *Bioinformatics Advances* online. Both approaches, based on individual omics data as well as the fusion/based approach were performed on four different models, including extreme gradient boosting (XGB), support vector classifier (SVC), multilayer perceptron (MLP), and TabNet. The fusion-based approach outperformed the models trained on individual omics datasets, including RNASeq (RNA) and copy number variation (CNV), in terms of accuracy, precision, specificity, and F1-score. The area under the receiver operator characteristic (AUROC) score, however, was observed to be relatively higher for XGB and SVC models when using RNASeq data alone. A similar trend was seen for recall and NPV metric when SVC model was trained on RNASeq data only. Finally, the AUROC was found slightly better for TabNet model when trained only on CNV, albeit for CNV data, the models did not produce effective AUROC score. The results indicate the importance of employing a fusion approach as relying on a single omics data may omit some crucial information that may prove effective for accurate classification. The value in bold represent the model achieving the maximum score in the underlying category. The subscript value represents the standard deviation. PPV: positive predicted values; NPV: negative predicted values.

**Figure 4:**
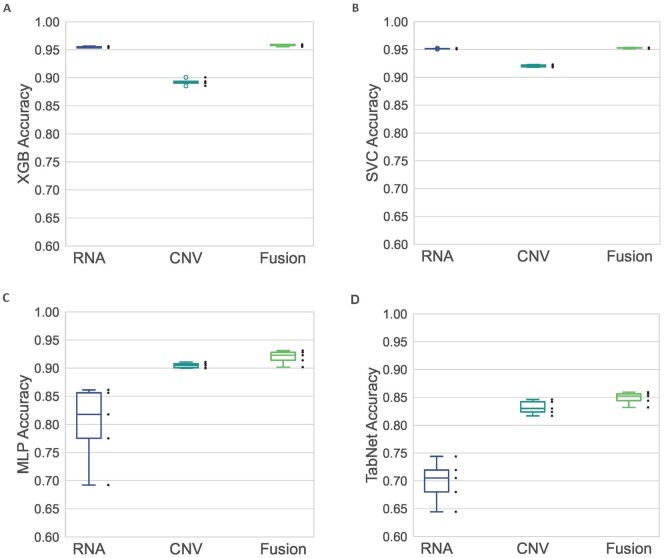
Accuracy achieved by each model when trained on individual omics dataset compared to the fusion-based approach. The box-and-whiskers plot summarizes the distribution of the accuracies achieved by each model across different random seed values. The bottom and the top of the box represents first and third quartiles, respectively, while the median is demonstrated by the line inside the box. Further, the whiskers represent the extreme values of the set (i.e. the minimum and maximum non-outlier values). The dots next to the boxes are the actual data points, representing the accuracy score. These box plots demonstrate that the proposed decision-level fusion mechanism surpasses all the models trained on individual omics datasets, including RNASeq (RNA) and copy number variation (CNV) data. Both approaches were employed on four different models: extreme gradient boosting (XGB; A), support vector classifier (SVC; B), multilayer perceptron (MLP; C) and TabNet (D). It should be noted that the ReliefF-based feature selection was performed on individual omics data after completion of the proposed fusion-based classification experiment. From a biological perspective, the improved performance of the fusion-based approach suggests that genomics and transcriptomics types provide complementary information, capturing distinct yet related molecular changes underlying NSCLC.

**Figure 5:**
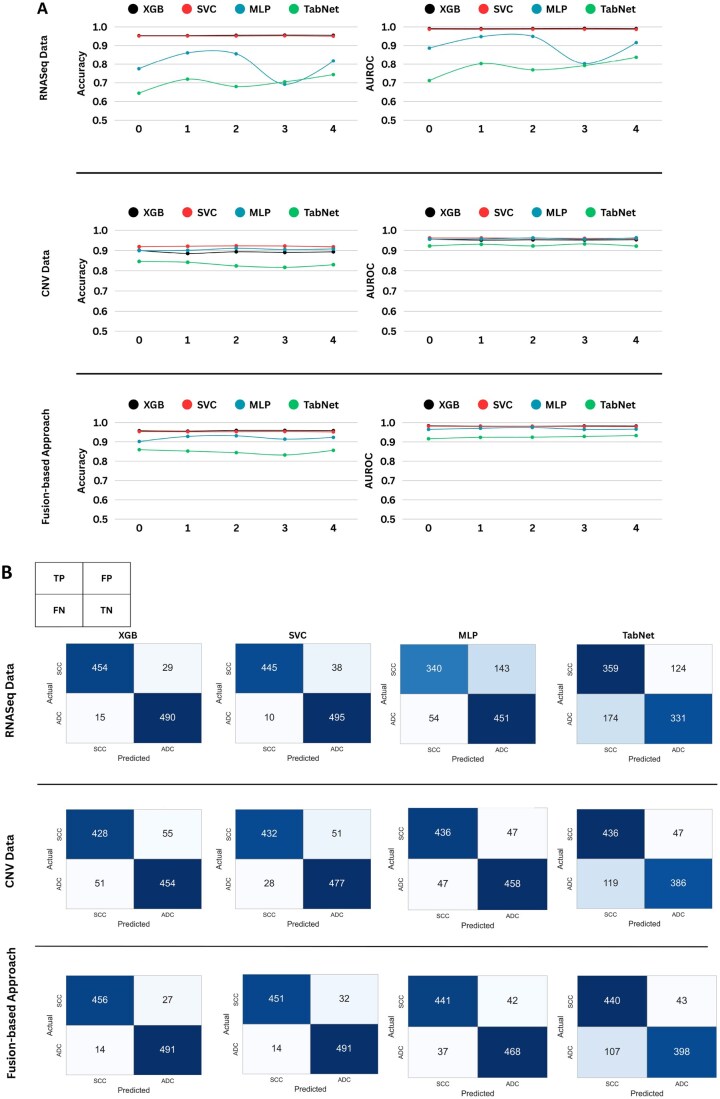
Line graphs and confusion matrices depicting the comparative prediction performance of models when trained on RNASeq (RNA) and copy number variation (CNV) data against the performance achieved when the fusion-based approach is employed. In the line graphs (A), the x-axis represents the five iterations conducted with random seed values, while the y-axis indicates the corresponding performance metrics, i.e. accuracy and AUROC scores. The confusion matrices (B) portray the absolute counts of TP, FP, FN and TN predictions made by each model, respectively, thus providing a comprehensive overview of their classification performance. Notably, the fusion-based approach exhibited superior performance compared to models trained on individual omics datasets, including RNASeq (RNA) and copy number variation (CNV), highlighting the advantage of integrating multiomics datasets. This finding applies to all the four utilized models: extreme gradient boosting (XGB), support vector classifier (SVC), multilayer perceptron (MLP) and TabNet. The improved performance of the fusion-based approach indicates that genomics copy number alterations and transcriptional changes capture complementary molecular processes within NSCLC; thus, their integration enables more accurate discrimination of disease-specific patterns.

Additionally, we found that the predictive performance across all models was improved in the fusion-based approach compared to the performance based on omics-discriminative models (Fig. 4). Further, Fig. 5 includes a line graph illustrating the accuracy and AUROC scores, alongside the corresponding confusion matrices, thus, presenting a comprehensive evaluation of model performance.

As observed, models trained on the individual omics datasets exhibited substantial variation in their predictive performance (Table 3 and Fig. 4). While XGB demonstrated high accuracy for RNASeq data (0.954±0.001), it performed worse on CNV data (0.892±0.005). A similar result was observed with the SVC model, which showed high accuracy on RNASeq data (0.951±0.0) but lower performance on CNV data (0.92±0.002). Interestingly, the MLP and TabNet models displayed an opposite outcome. The MLP model achieved an accuracy of 0.80±0.069 on RNASeq and performed better on CNV data (0.904±0.004). Similarly, the TabNet model achieved an accuracy of 0.698±0.038 on RNASeq and better accuracy (0.831±0.012) on CNV data.

However, the decision-level fusion strategy demonstrated a consistent superior classification accuracy (0.958±0.001 using XGB, 0.952±0.0 using SVC, 0.919±0.011 using MLP and 0.848±0.01 using TabNet) compared to all the models trained on individual omics datasets, thus, relatively mitigating the high variation in the models. Based on these observations, it could be inferred that relying on single omics data may lead to varying predictions that do not identify a concise selection of pathologically relevant key genes.

### 3.2 ReliefF-derived key NSCLC-relevant genes

The utility of ReliefF algorithm for gene discovery resulted in an unveiling of a concise set of 47 most relevant NSCLC genes that are consistent across both the omics datasets—transcriptomics and genomics. The complete list of genes is presented in Table 4. Further, we performed Pearson’s correlation analysis to compute pairwise correlations among the identified set of 47 genes (see Fig. 2, available as supplementary data at *Bioinformatics Advances* online). It could be confirmed via a literature search that 39 of the identified genes were already associated with lung cancer by either a biological or a computational analysis, which supports the reliability of our fusion-based approach to identify a focused set of key genes. Among the 47 found genes, we discovered a total of seven genes, namely *ABCF3* (Fan *et al.* 2023), *ACAP2* (Liu *et al.* 2022), *LSG1* (Shen *et al.* 2022), *TBCCD1* (Denu and Burkard 2020), *UCN2* (Hao *et al.* 2008), *WDR53* (Weissmiller *et al.* 2024) and *ZNF639* (Imoto *et al.* 2003), that could play a role in NSCLC but have only been investigated in the context of other cancer types until now. Another gene, *FYTTD1*, was found as a gene in our analysis which is currently only vaguely studied in pathologies without any indication for its role in any type of cancer yet.

**Table 4:** The list of predictive genes.^a^

*ABCC5*	*ABCF3*	*ACAP2*	*ACTL6A*	*AP2M1*
*ATP11B*	*CLDN1*	*COL7A1*	*DCUN1D1*	*DLG1*
*DVL3*	*FBXO45*	*FXR1*	*FYTTD1*	*GMPS*
*KPNA4*	*LRRC31*	*LSG1*	*MED12L*	*MFN1*
*MYNN*	*OPA1*	*P2RY1*	*PAK2*	*PARL*
*PCYT1A*	*PDCD10*	*PIGX*	*PIK3CA*	*PLCH1*
*PLD1*	*PSMD2*	*RFC4*	*RNF168*	*RSRC1*
*SEMA3F*	*SENP5*	*SIAH2*	*SOX2*	*TBCCD1*
*TFRC*	*TP63*	*TRA2B*	*UCN2*	*WDR53*
*YEATS2*	*ZNF639*			

a The following list comprises the discovered NSCLC-relevant 47 genes using the ReliefF method.

### 3.3 Predictive performance of key NSCLC-relevant genes

Table 5 presents the classification performance of the identified set of 47 NSCLC-relevant genes on the CPTAC validation cohort. The evaluation metrics employed were accuracy and AUROC scores. The XGB model exhibited a classification accuracy of 0.879±0.008,0.835±0.002 and 0.912±0.013 for transcriptomics, genomics and fusion-based approach, respectively. The results indicate strong predictive power of the identified genes; however, since the external validation was limited to a single independent dataset (CPTAC), further validation across additional independent cohorts would be valuable to comprehensively establish the generalizability of the discovered genes.

**Table 5:** Predictive performance of the identified set of 47 genes on independent dataset (mean ± standard deviation).^a^

Dataset/approach	Accuracy	AUROC
Transcriptomics	0.879${}_{\pm 0.008}$	0.948${}_{\pm 0.010}$
Genomics	0.835${}_{\pm 0.002}$	0.860${}_{\pm 0.004}$
**Proposed fusion approach**	**0.912** ${}_{\pm 0.013}$	**0.979** ${}_{\pm 0.005}$

a We employed the extreme gradient boosting (XGB) model for validation purpose. The results demonstrate that the proposed fusion-based approach outperforms the models trained on individual omics types, achieving superior accuracy and AUROC scores. These findings indicate that the fusion-based strategy is robust and capable of delivering accurate predictions on unseen datasets. The text in bold represents the model achieving the maximum score in the underlying category. The subscript represent the standard deviation.

#### 3.3.1 SHAPley-based attribution of identified genes

**Figure 6:**
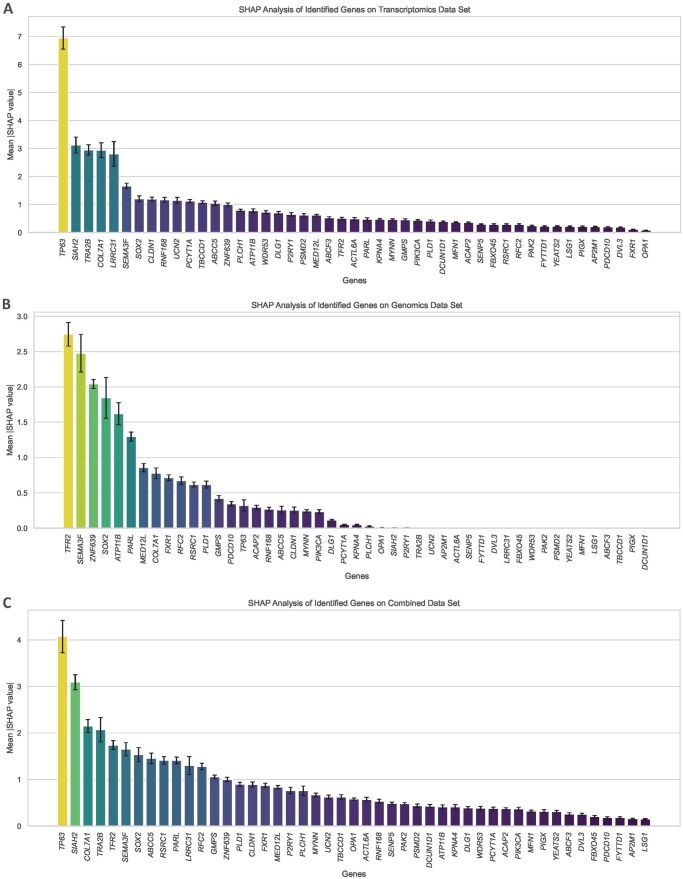
SHAP analysis of the contribution of identified genes in subtype classification. The x-axes of the bar plots indicate the identified set of genes, while the y-axes represent the mean absolute SHAP score, i.e. the contribution of each gene toward NSCLC subtype classification. Error bars represent the 95% confidence intervals of mean SHAP values across independent experimental runs. The plots illustrate SHAP analysis when the XGB model was trained on (A) CPTAC RNASeq transcriptomics dataset; (B) CPTAC CNV genomics dataset; and (C) combined dataset. The analysis unveils the top genes such as *TP63, ABCC5, SOX2, DCUN1D1* and *PIK3CA*, that are well-established in case of NSCLC and are associated with various cancer-related processes, such as tumor differentiation, cell cycle regulation, DNA damage response and oncogenic signaling pathways, thus supporting the biological plausibility and consistency with the known NSCLC biological pathways.

Figure 6 illustrates the individual attribution of each of the 47 identified genes (in terms of contribution score) toward classification when utilized only with transcriptomics data, genomics data and when both the omics datasets were combined.

### 3.4 Enrichment analysis of discovered genes

**Figure 7:**
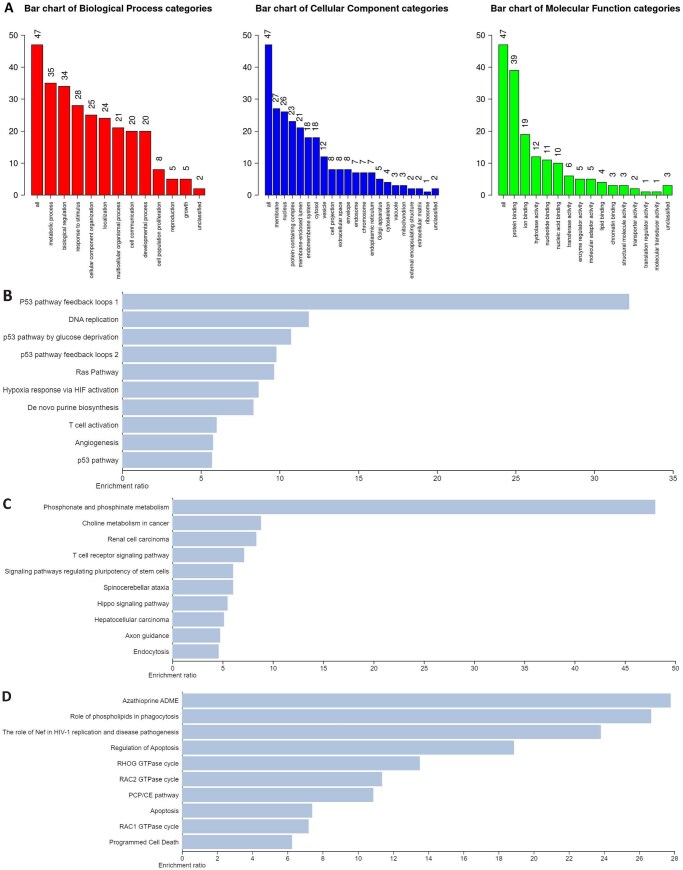
Enrichment analysis of the 47 discovered genes. The discovered genes are associated with a variety of biological processes, cellular components and molecular functions (A). The bar plots illustrate the functional pathways enriched in the 47 discovered genes found in the PANTHER (B), KEGG (C) and Reactome databases (D), respectively. The *x*-axis represents the enrichment ratio and the *y*-axis lists the distinct biological pathways. The enrichment ratio indicates the degree of association of the genes with the respective pathway. Notably, many of the enriched processes and pathways are consistent with established NSCLC analyses, including cancer-related signaling, cell proliferation and regulatory mechanisms, supporting the biological relevance of the identified gene set.

The discovered genes were found significantly associated with numerous biological processes (Fig. 7), including *metabolic process, biological regulation, response to stimulus, cellular component organization, localization, multicellular organismal process, cell communication* and *developmental process*. The cellular components found enriched were *membrane, nucleus, protein-containing complex, membrane-enclosed lumen, endomembrane system, cytosol* and *vesicle*. Among the molecular functions, the protein binding, ion binding, hydrolase activity, nucleotide binding and nucleic acid binding were found enriched.

Among the functional pathways, the *Ras pathway* (*P* = .002), *angiogenesis* (*P* = .003), *p53 pathway feedback loops 2* (*P* = .016), *p53 pathway feedback loops 1* (*P* = .030), *T cell activation* (*P* = .041) and *p53 pathway* (*P* = .045) were found to be significantly enriched in the PANTHER database. The KEGG database showed the *choline metabolism in cancer* (*P* = .004), *T cell receptor signaling pathway* (*P* = .008), *endocytosis* (*P* = .0010), *signaling pathways regulating pluripotency of stem cells* (*P* = .012), *spinocerebellar ataxia* (*P* = .012), *hippo signaling pathway* (*P* = .016), *hepatocellular carcinoma* (*P* = .019), *phosphonate and phosphinate metabolism* (*P* = .020), *renal cell carcinoma* (*P* = .023) and *axon guidance* (*P* = .024) pathways to be significantly enriched. Finally, the *regulation of apoptosis* (*P* = .005), *RHOG GTPase cycle* (*P* = .001), *apoptosis* (*P* = .001), *RAC1 GTPase cycle* (*P* = .002), *RAC2 GTPase cycle* (*P* = .002), *azathioprine ADME* (*P* = .002), *role of phospholipids in phagocytosis* (*P* = .002), *PCP/CE pathway* (*P* = .002), *the role of Nef in HIV-1 replication and disease pathogenesis* (*P* = .003) and *programmed cell death* (p = 0.003) were the pathways found enriched in the Reactome database.

### 3.5 Overall survival analysis of the discovered genes

**Figure 8:**
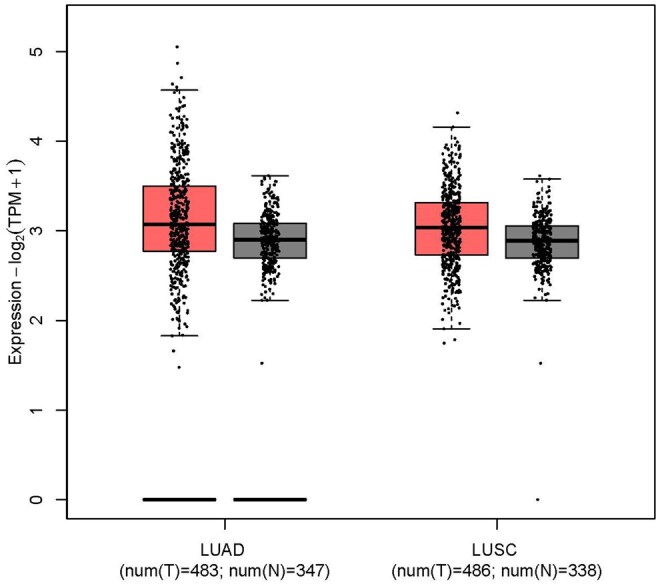
Gene expression-based boxplot of combined expression values of *PLCH1* (*KIAA1069*), *LRRC31*, *RNF168* and *TRA2B* genes. The plot summarizes the distribution of the data samples. The bottom and the top of the box represents first and third quartiles, respectively, while the median is demonstrated by the line inside the box. Further, the whiskers represent the extreme values of the set (i.e. the minimum and maximum non-outlier values). The dots across the boxes are the actual sample points, while the dots beyond the whiskers are considered outliers. This analysis revealed that the expression of these genes was relatively lower in normal samples as compared to the tumorous samples, thus, supporting their prognostic relevance in the non-small cell lung cancer subtypes lung adenocarcinoma (LUAD) and lung squamous cell carcinoma (LUSC).

The evaluation of the prognostic efficacy of the discovered set of 47 genes unveiled a subset of 28 genes that demonstrated significant prognostic potential with *P*≤.05. The KM curves of these 28 genes are in Fig. 3, available as supplementary data at *Bioinformatics Advances* online. It is observed that except for *PLCH1 (KIAA1069), LRRC31, RNF168* and *TRA2B*, the lower expression values of the remaining 24 genes were associated with poor prognosis. To further validate this observation, we investigated the combined expression levels of *PLCH1 (KIAA1069), LRRC31, RNF168* and *TRA2B* in healthy and tumor samples using data from TCGA and Genotype-Tissue Expression (GTEx) via the Gene Expression Profiling Interactive Analysis (GEPIA2) platform (Tang *et al.* 2019). The platform provided a comprehensive dataset of ADC and SCC subtypes, comprising 483 tumors and 347 normal samples in the ADC cohort as well as 486 tumors and 338 normal samples in the SCC cohort. A similar trend was observed, showing lower expression levels of the aforementioned four genes in normal samples as compared to the tumorous samples (Fig. 8).

### 3.6 Comparison with state-of-the-art

The prediction performance of the proposed decision-level fusion approach was benchmarked against the state-of-the-art literature. Table 6 presents the comparison—*Carrillo-Perez et al.* for multiomics data, *Girard et al.* for the RNASeq data and *Qiu et al.* for the CNV data. The proposed approach outperformed all the aforementioned works, underscoring the utility of multiomics integration over single omics models for NSCLC subtype prediction.

**Table 6:** Comparison with related research works.^a^

	Fusion-based	RNASeq	CNV
*Carrillo-Perez et al.*	0.958	0.950	**0.969**
*Girard et al.*	–	0.950	–
*Qiu et al.*	–	–	0.840
*Dwivedi et al.*	–	–	0.849
**Proposed approach**	**0.958** ${}_{\pm 0.001}$	**0.954** ${}_{\pm 0.001}$	0.892${}_{\pm 0.005}$

a The proposed weighted-average-based decision-level fusion approach was found to be competitive with the established researches in case of multiomics integration and RNASeq gene expression omics data. The accuracy is presented in (mean ± standard deviation) form. The values in bold represent the model/approach achieving maximum score in the underlying category. The subscripts represent the standard deviation. (It should be noted that a direct comparison between the results shown is not feasible as the train and validation data split of the related works were not known.)

## 4 Discussion

The primary objective of the proposed study was to identify a highly accurate approach for the discovery of key NSCLC genes that could assist in improving targeted therapeutic strategies in future. To achieve this, multiple omics data were utilized in an integrative manner to capture the complex interconnected biological dependencies underlying NSCLC subtypes.

Our previous studies (Dwivedi *et al.* 2023, 2024a, 2024b) primarily focused on analyzing individual omics modalities, in particular transcriptomics, genomics and epigenomics data. Although Dwivedi *et al.* (2023) and Dwivedi *et al.* (2024a) explored multiomics integration, their methodology employed a direct feature-level concatenation of different omics modalities. In contrast, this study introduced a multiomics approach to genes identification by employing a weighted-average-based decision-level fusion mechanism that fused the predicted probabilities generated by ML models trained on individual omics datasets. Initially, the classification performance of individual omics datasets was compared with that of the fusion-based approach by employing various evaluation metrics. As hypothesized, the weighted-average-based decision-level-fusion approach demonstrated superior prediction performance compared to the models trained on individual omics datasets. Thereafter, the identification of a set of NSCLC-relevant genes that are cohesively responsible for accurate subtype classification was performed and unveiled a set of 47 key genes, which play a role in different aspects and stages of NSCLC development (Table 4).

In addition to the comparison with models trained on individual omics data, the proposed weighted-average-based decision-level fusion method was compared with competing approaches, to provide a contrastive view of the fusion-based concept. In particular, we compared the proposed strategy with standard ensemble techniques such as hard voting and soft voting schemes (Table 7). These conventional voting strategies assign equal weight to predictions from individual modality, irrespective of their “*predictive confidence*” (predictive probability score). In contrast, the proposed fusion-based approach assigns differential weights to the predictions generated by individual omics models based on their relative importance, enabling a “*confidence-derived*” integration of complementary cross-modality information.

**Table 7:** Results of ablation studies (mean ± standard deviation).^a^

#Study	Model-XGB/approach	**Accuracy**		**AUROC**	
**1.**	Joint Omics	0.922${}_{\pm 0.002}$		0.973${}_{\pm 0.000}$	
	Fusion-based	**0.958** ${}_{\pm 0.001}$		0.982${}_{\pm 0.001}$	
**2.**	Hard Voting	0.926${}_{\pm 0.005}$		–	
	Soft Voting	0.954${}_{\pm 0.002}$		–	
	RNASeq	0.954${}_{\pm 0.001}$		–	
	CNV	0.892${}_{\pm 0.005}$		–	
	Fusion-based	**0.958** ${}_{\pm 0.001}$		–	

		**Union-derived Genes (931)**.	**Intersection-derived Genes (47)**.	**Union-derived Genes (931)**.	**Intersection-derived Genes (47)**.

**3.**	RNASeq	**0.957** ${}_{\pm 0.002}$	**0.936** ${}_{\pm 0.001}$	**0.988** ${}_{\pm 0.000}$	**0.978** ${}_{\pm 0.002}$
	CNV	0.889${}_{\pm 0.002}$	0.818${}_{\pm 0.001}$	0.950${}_{\pm 0.002}$	0.887${}_{\pm 0.000}$
	Fusion-based	0.947${}_{\pm 0.000}$	0.934${}_{\pm 0.000}$	0.978${}_{\pm 0.001}$	0.960${}_{\pm 0.000}$

a All the studies were performed using the XGB model on TCGA cohorts. (1) We utilized the joint dataset comprising both genomics and transcriptomics samples and features sets. (2) The proposed weighted-average-based decision-level fusion approach was compared with the standard ensemble techniques, including hard and soft voting mechanisms. (3) We computed the union of NSCLC-relevant genes identified by ReliefF method for both genomics and transcriptomics data. The predication performance of union-derived genes was subsequently compared with that of the intersection-derived genes. Although the union-derived genes exhibited relatively better performance than the intersection-derived genes, it should be noted that the number of features set increased manifolds (931 union-derived compared to 47 intersection-derived genes). The values in bold represent the model/approach achieving maximum score in the underlying category. The subscripts represent the standard deviation.

From a biological perspective, it is important to understand that RNASeq and CNV data capture different but mechanistically linked layers of gene regulation. CNV data represent structural changes at DNA level occurring from, e.g. duplication or deletion of base pairs and thus affecting the genetic dosage that on the one side ensures sufficient biological variation among a population but on the other side also gives rise to disease phenotypes. However, CNV data alone do not necessarily imply any functional consequences as changes in copy numbers can be balanced by transcriptional or post-transcriptional processes. Hence, RNA-seq data are needed to reveal transcriptomic activity. Therefore, our multi-omics approach does not only outperform single-omics approaches in accuracy but also provides deeper biological insights as, e.g. parallel CNV gain and RNA overexpression or CNV loss and reduced RNA expression point to strong candidates for tumor pathway regulation. Conversely, RNA expression changes without corresponding CNV alteration and CNV changes without RNA expression changes rather suggest involvement in compensatory or other signaling mechanisms. This work therefore reveals a novel set of candidate genes for NSCLC regulation whose expression is both structurally and transcriptionally supported, highlighting molecular drivers that would remain obscured when analyzing CNV or transcriptomic profiles only.

While the publicly available datasets such as TCGA are comprehensive in nature, their employment is subject to an important limitation, as they may introduce inherent biases due to the patient demographics, history of treatment and medication as well as methodology followed to collect the data (Dwivedi *et al.* 2024a). Another potential limitation when handling omics data with ML techniques is the *curse of dimensionality*, i.e. datasets following high-dimension-low-sample-size (HDLSS) format with number of patient samples relatively lower than the number of genes. As machine/deep learning models have high capacity to learn complex, entangled relationships, the HDLSS datasets may introduce overfitting and thus may lead to weaker generalization of the model and instability in feature selection (Ng *et al.* 2023, Wysocka *et al.* 2023).

## 5 Conclusion

This study hypothesized that NSCLC subtype prediction can be improved by using a fusion of multiomics data in contrast to relying on an individual omics type. To validate, we developed a weighted-average-based decision-level fusion mechanism to fuse gene expression and CNV omics data. The employed approach resulted in a superior subtype classification accuracy when compared to individual omics-based predictive models, confirming that the proposed hypothesis holds true. Subsequently, as the key output of the experiment, we identified a concise set of NSCLC-relevant genes and conformed their biological significance through functional pathways and survival analyses. As a future work, the proposed fusion-based approach may be enhanced to develop a deep learning-based foundation model by incorporating large-scale omics data along with different modalities such as image or clinical information of the patients. This multi-modal foundation model would have a potential to significantly improve overall classification performance and assist in a more robust identification of molecular targets that may further be evaluated to determine their diagnostic and prognostic significance. The study also establishes a foundation for future targeted experimental studies, where the identified genes could be validated quantitatively and mechanistically to strengthen their biological and functional relevance.

## Supplementary Material

vbag131_Supplementary_Data

## Data Availability

Data and source code are available on: https://github.com/kountaydwivedi/multiomics_fusion.git.

## Appendix

### Ablation studies

We performed the following ablation studies to compare with the proposed fusion-based approach:

1. Joint model combining transcriptomics and genomics features
2. Fusion of omics data using standard ensemble techniques including hard and soft voting
3. Union of NSCLC-relevant genomics and transcriptomics genes derived using ReliefF method.

The results for all the ablation study are presented in Table 7. Each study was performed on the TCGA cohort, trained and validated using stratified *k*-fold cross validation (*k *= 5) technique on XGB model. For robust and stable comparison, each experiment was repeated five times, each time with a different random seed value.

A brief description of the mathematical formulation of hard and soft voting is as follows:

- **Hard voting**: Often known as majority voting, the hard voting mechanism determines the class label of a sample based on majority of votes among all the datasets. Mathematically, we implemented hard voting as follows: Assume f(·) to be a predictor and *i* to be a sample, then:
  (A.1) $$C_{i}^{\mathrm{rna}}:=f(i_{\mathrm{RNASeq}})$$
  (A.2) $$C_{i}^{\mathrm{cnv}}:=f(i_{\mathrm{CNV}})$$

where $C_{i}^{\mathrm{rna}}$ and $C_{i}^{\mathrm{cnv}}$ are the predicted class labels (ADC or SCC) of *i* in RNASeq and CNV datasets, respectively. Subsequently, the final prediction for *i* is computed as:

(A.3)
$${\tilde{y}}_{i}:=C_{i}^{\mathrm{rna}}\vee C_{i}^{\mathrm{cnv}}$$

where ∨ is the binary-OR.

- **Soft voting**: Unlike hard voting, the soft voting mechanism aggregates the prediction probabilities among all the datasets. Specifically, for a sample *i* and predictor f(·):
  (A.4) $$P_{i}^{\mathrm{rna}}:=\mathrm{Pro}b_{f(i_{\mathrm{RNASeq}})}$$
  (A.5) $$P_{i}^{\mathrm{cnv}}:=\mathrm{Pro}b_{f(i_{\mathrm{CNV}})}$$

where $P_{i}^{\mathrm{rna}}$ and $P_{i}^{\mathrm{cnv}}$ are the predicted probabilities of *i* in RNASeq and CNV datasets, respectively. Here, the final prediction for *i* is computed as follows:

(A.6)
$$\mathrm{SoftPro}b_{\mathrm{SCC}}:=((1-P_{i}^{\mathrm{rna}})+(1-P_{i}^{\mathrm{cnv}}))/2$$

(A.7)
$$\mathrm{SoftPro}b_{\mathrm{ACC}}:=(P_{i}^{\mathrm{rna}}+P_{i}^{\mathrm{cnv}})/2$$

(A.8)
$${\tilde{y}}_{i}:=\{\begin{array}{c} 1\;\mathrm{pro}b_{\mathrm{ADC}}\geq \mathrm{pro}b_{\mathrm{SCC}}\\ 0\;\mathrm{otherwise} \end{array}$$

Notably, all the employed models along with the proposed weighted-average-based decision-level fusion technique performed superior to when the joint genomics and transcriptomics datasets were utilized for NSCLC subtype classification. Further, the standard hard and soft voting mechanism also exhibited inferior performance compared to the proposed approach, although the hard and soft voting mechanisms outperformed the model trained on the genomics data alone with over 3% and 6% accuracy, respectively.

When we took the union of the NSCLC-relevant genomics and transcriptomics genes (using ReliefF method), the entire feature set was extended from *intersection-derived* 47 genes to *union-derived* 931 genes. The *union-derived* genes, although demonstrated slightly better predictive performance than the *intersection-derived* genes, the gain was not substantial enough to justify the considerably larger number of genes required. Thus, the marginal improvement in accuracy should be interpreted with caution given the increased model complexity introduced by the large gene set.
