# Supplementary material for: A fusion-based multiomics classification approach for enhanced gene discovery in non-small cell lung cancer
Source: Bioinform Adv. 2026 May 11;6(1):vbag131. doi: 10.1093/bioadv/vbag131 (PMC13242183; doi:10.1093/bioadv/vbag131)
Supplement: vbag131_Supplementary_Data [file vbag131_supplementary_data.pdf]

Bioinformatics Advances, , pp. 1–6

Supplementary Article

Supplementary Article:A Fusion-Based Multiomics Classification Approach for Enhanced Gene Discovery in Non-Small Cell Lung Cancer

Kountay Dwivedi ,[1] Amirreza Mahbod ,[2] Rupert C. Ecker [3] and Klara Janjić [4,*]

[1]Center for Clinical Research, University Clinic of Dentistry, Medical University of Vienna, Vienna, Austria

[2]Research Center for Medical Image Analysis and Artificial Intelligence, Department of Medicine, Faculty of Medicine and Dentistry, Danube Private University, 3500 Krems an der Donau, Austria

[3]TissueGnostics GmbH, 1020 Vienna, Austria

[*]Corresponding author. klara.janjic@meduniwien.ac.at

Abstract

Motivation: This study introduces a fusion-based multiomics approach to identifying non-small cell lung cancer (NSCLC)-relevant genes. We evaluated the NSCLC-subtype classification performance of various state-of-the-art machine learning models using single omics and fused multiomics approaches. The models were trained separately on individual omics data sets. Subsequently, a weighted-average-based decision-level fusion mechanism was employed to integrate the individual predictions of the trained models. Finally, the prediction performance across all the approaches was compared.

Results: The decision-level fusion-based approach yielded a superior classification performance as compared to the performance achieved by models trained on individual omics data sets. Finally, a set of 47 NSCLC-relevant genes were identified. For the first time, ABCF3, ACAP2, LSG1, TBCCD1, UCN2, WDR53, ZNF639 and FYTTD1 appeared in the context of NSCLC. In conclusion, the integration of multiple omics types showed potential to deliver a more concise selection of NSCLC-relevant genes that could be clinically targeted in future.

Availability: Data and source code are available on: https://github.com/kountaydwivedi/multiomics_fusion.git

Contact: klara.janjic@meduniwien.ac.at

Keywords Computational biology, Multi-omics, Cancer, Transcriptomics, Genomics

© The Author(s) . Copyright and licence statements to be updated by the publisher during production.

Supplementary Table 1. Summary of the demographic and clinical attributes of the TCGA lung cancer cohort, stratified by non-small cell lung cancer (NSCLC) subtype (lung adenocarcinoma, ADC; lung squamous cell carcinoma, SCC) and omics types (RNASeq transcriptomics and copy number variation (CNV) genomics).

**Attribute**	**Lung Adenocarcinoma (ADC)**		**Lung Squamous Cell Carcinoma (SCC)**	
	**RNASeq Transcriptomics**	**CNV Genomics**	**RNASeq Transcriptomics**	**CNV Genomics**
**Age**	38-88	33-88	39-90	39-90
**Overall Survival**	Living: 325<br>Dead: 185	Living: 328<br>Dead: 183	Living: 207<br>Dead: 277	Living: 210<br>Dead: 277
**Gender**	Male: 236<br>Female: 274	Male: 238<br>Female: 273	Male: 356<br>Female: 126	Male: 358<br>Female: 127
**Tumor Stage**	NA: 2<br>Stage I: 5<br>Stage IA: 132<br>Stage IB: 138<br>Stage II: 1<br>Stage IIA: 52<br>Stage IIB: 70<br>Stage IIIA: 72<br>Stage IIIB: 11<br>Stage IV: 27	NA: 2<br>Stage I: 5<br>Stage IA: 134<br>Stage IB: 138<br>Stage II: 1<br>Stage IIA: 52<br>Stage IIB: 71<br>Stage IIIA: 71<br>Stage IIIB: 11<br>Stage IV: 2	NA: 3<br>Stage I: 2<br>Stage IA: 85<br>Stage IB: 148<br>Stage II: 2<br>Stage IIA: 62<br>Stage IIB: 93<br>Stage III: 3<br>Stage IIIA: 61<br>Stage IIIB: 18<br>Stage IV: 7	NA: 3<br>Stage I: 2<br>Stage IA: 85<br>Stage IB: 149<br>Stage II: 2<br>Stage IIA: 62<br>Stage IIB: 94<br>Stage III: 3<br>Stage IIIA: 61<br>Stage IIIB: 19<br>Stage IV: 7
**Ethnicity**	Hispanic/Latino: 7<br>NA: 124<br>Not Hispanic/Latino: 379	Hispanic/Latino: 7<br>NA: 124<br>Not Hispanic/Latino: 380	Hispanic/Latino: 8<br>NA: 175<br>Not Hispanic/Latino: 301	Hispanic/Latino: 8<br>NA: 175<br>Not Hispanic/Latino: 304
**Biogeographic Origin**	American Indian or Alaska Native: 1<br>Asian: 8<br>Black or African American: 52<br>NA: 65<br>White: 384	American Indian or Alaska Native: 1<br>Asian: 7<br>Black or African American: 52<br>NA: 65<br>White: 386	American Indian or Alaska Native: 1<br>Asian: 8<br>Black or African American: 52<br>NA: 65<br>White: 384	Asian: 9<br>Black or African American: 29<br>NA: 112<br>White: 337

Supplementary Table 2.: Prediction performance of each individual omics data set alongside fusion-based approach (mean ± standard deviation). Values in square brackets [] indicate 95% confidence intervals computed across five independent runs. Both approaches, based on individual omics data as well as the fusion/based approach were performed on four different models, including Extreme Gradient Boosting (XGB), Support Vector Classifier (SVC), Multilayer Perceptron (MLP) and TabNet. The fusion-based approach outperformed the models trained on individual omics data sets, RNASeq (RNA) and copy number variation (CNV) in terms of accuracy, precision, specificity and F1-score. The area under the receiver operator characteristic (AUROC) score, however, was observed to be relatively higher for XGB and SVC models when using RNASeq data alone. A similar trend was seen for recall and NPV metric when SVC model was trained on RNASeq data only. Finally, the AUROC was found slightly better for TabNet model when trained only on CNV, albeit for CNV data, the models did not produce effective AUROC score. The results indicate the importance of employing a fusion approach as relying on a single omics data may omit some crucial information that may prove effective for accurate classification.

Model/Approach	Accuracy	AUROC	Precision (PPV)	Recall (Sensitivity)	Specificity	F1-Score	NPV
XGB-RNA	0.954±0.001 [0.953–0.955]	0.991±0.000 [0.991–0.991]	0.942±0.002 [0.940–0.944]	0.970±0.001 [0.969–0.971]	0.938±0.002 [0.936–0.940]	0.956±0.001 [0.955–0.957]	0.968±0.001 [0.967–0.969]
XGB-CNV	0.892±0.005 [0.886–0.898]	0.953±0.001 [0.952–0.954]	0.891±0.003 [0.887–0.895]	0.899±0.008 [0.889–0.909]	0.885±0.003 [0.881–0.889]	0.895±0.005 [0.889–0.901]	0.894±0.008 [0.884–0.904]
**XGB-Fusion**	0.958±0.001 [0.957–0.959]	0.982±0.001 [0.981–0.983]	0.947±0.002 [0.945–0.949]	0.972±0.003 [0.968–0.976]	0.943±0.003 [0.939–0.947]	0.959±0.001 [0.958–0.960]	0.970±0.003 [0.966–0.974]
SVC-RNA	0.951±0.000 [0.951–0.951]	0.987±0.000 [0.987–0.987]	0.928±0.001 [0.927–0.929]	0.980±0.000 [0.980–0.980]	0.921±0.001 [0.920–0.922]	0.954±0.000 [0.954–0.954]	0.977±0.000 [0.977–0.977]
SVC-CNV	0.920±0.002 [0.918–0.922]	0.960±0.000 [0.960–0.960]	0.900±0.003 [0.896–0.904]	0.944±0.001 [0.943–0.945]	0.894±0.003 [0.890–0.898]	0.924±0.001 [0.923–0.925]	0.939±0.001 [0.938–0.940]
**SVC-Fusion**	0.952±0.000 [0.952–0.952]	0.980±0.000 [0.980–0.980]	0.938±0.001 [0.937–0.939]	0.971±0.001 [0.970–0.972]	0.932±0.002 [0.930–0.934]	0.954±0.000 [0.954–0.954]	0.969±0.001 [0.968–0.970]
MLP-RNA	0.800±0.069 [0.714–0.886]	0.900±0.060 [0.825–0.975]	0.786±0.125 [0.631–0.941]	0.893±0.117 [0.748–1.038]	0.703±0.230 [0.417–0.989]	0.822±0.042 [0.770–0.874]	0.892±0.095 [0.774–1.010]
MLP-CNV	0.904±0.004 [0.899–0.909]	0.958±0.003 [0.954–0.962]	0.907±0.004 [0.902–0.912]	0.906±0.008 [0.896–0.916]	0.902±0.005 [0.896–0.908]	0.907±0.004 [0.902–0.912]	0.902±0.007 [0.893–0.911]
**MLP-Fusion**	0.919±0.011 [0.905–0.933]	0.968±0.004 [0.963–0.973]	0.917±0.006 [0.910–0.924]	0.926±0.019 [0.902–0.950]	0.912±0.006 [0.905–0.919]	0.921±0.012 [0.906–0.936]	0.922±0.019 [0.898–0.946]
TabNet-RNA	0.698±0.038 [0.651–0.745]	0.782±0.046 [0.725–0.839]	0.733±0.065 [0.652–0.814]	0.655±0.048 [0.595–0.715]	0.743±0.084 [0.639–0.847]	0.690±0.032 [0.650–0.730]	0.673±0.030 [0.636–0.710]
TabNet-CNV	0.831±0.012 [0.816–0.846]	0.926±0.005 [0.920–0.932]	0.891±0.015 [0.872–0.910]	0.764±0.038 [0.717–0.811]	0.902±0.020 [0.877–0.927]	0.823±0.016 [0.803–0.843]	0.786±0.024 [0.756–0.816]
**TabNet-Fusion**	0.848±0.010 [0.836–0.860]	0.925±0.006 [0.918–0.932]	0.903±0.017 [0.882–0.924]	0.788±0.032 [0.748–0.828]	0.911±0.020 [0.886–0.936]	0.841±0.014 [0.824–0.858]	0.805±0.021 [0.779–0.831]

Supplementary Table 3. Statistical-based comparison of the prediction performance of individual omics datasets with the fusion-based approach. The Mann Whitney-U test was utilized to compare the accuracy and AUROC scores of each model across the five independent iterations using different random seed values. The Mann Whitney U-test requires two lists of values for analysis. In this case, the first and second list was the metric values obtained using individual omics data and fusion-based approach, respectively. This was done for both metrics—accuracy and AUROC score—for each model. The test of significance was carried out by analysing the p-value, while the effect size was reported using ran-biserial correlation method. p-value $\leq$ 0.05 indicates statistical significance of the proposed hypothesis against the null hypothesis, while effect size indicates the magnitude of the clinical relevance of the findings. In our case, since the number of random iterations of the experiment was five, which represents the minimum sample size to perform Mann-Whitney U-test, we did not apply multiple-testing correction to the obtained p-values.

**Model**	**Accuracy**		**AUROC**	
	**Transcriptomics <->Fusion**	**Genomics <->Fusion**	**Transcriptomics <->Fusion**	**Genomics <->Fusion**
**XGB**	p-value: 0.033<br>effect-size: 0.84	p-value: 0.011<br>effect-size: 1.0	p-value: 0.007<br>effect-size: -1.0	p-value: 0.007<br>effect-size: 1.0
**SVC**	p-value: 0.062<br>effect-size: 0.72	p-value: 0.011<br>effect-size: 1.0	p-value: 0.007<br>effect-size: -1.0	p-value: 0.007<br>effect-size: 1.0
**MLP**	p-value: 0.007<br>effect-size: 1.0	p-value: 0.055<br>effect-size: 0.76	p-value: 0.007<br>effect-size: 1.0	p-value: 0.007<br>effect-size: 1.0
**TabNet**	p-value: 0.007<br>effect-size: 1.0	p-value: 0.055<br>effect-size: 0.76	p-value: 0.033<br>effect-size: 0.84	p-value: 0.841<br>effect-size: 0.12

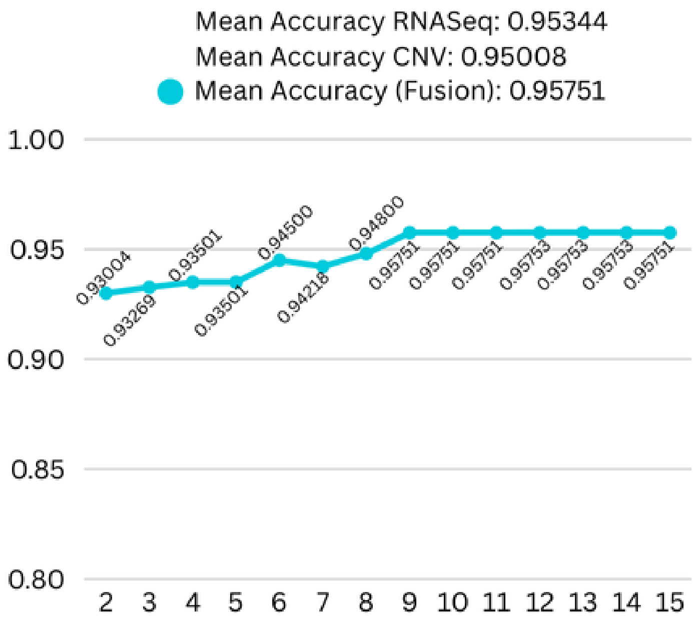

Supplementary Figure 1. Plot illustrating the variation in accuracy (y-axis) achieved by fusion-based approach with different values of k (x-axis). The fusion-based accuracy showed gradual improvement and eventually flattened when k $\geq$ 10.

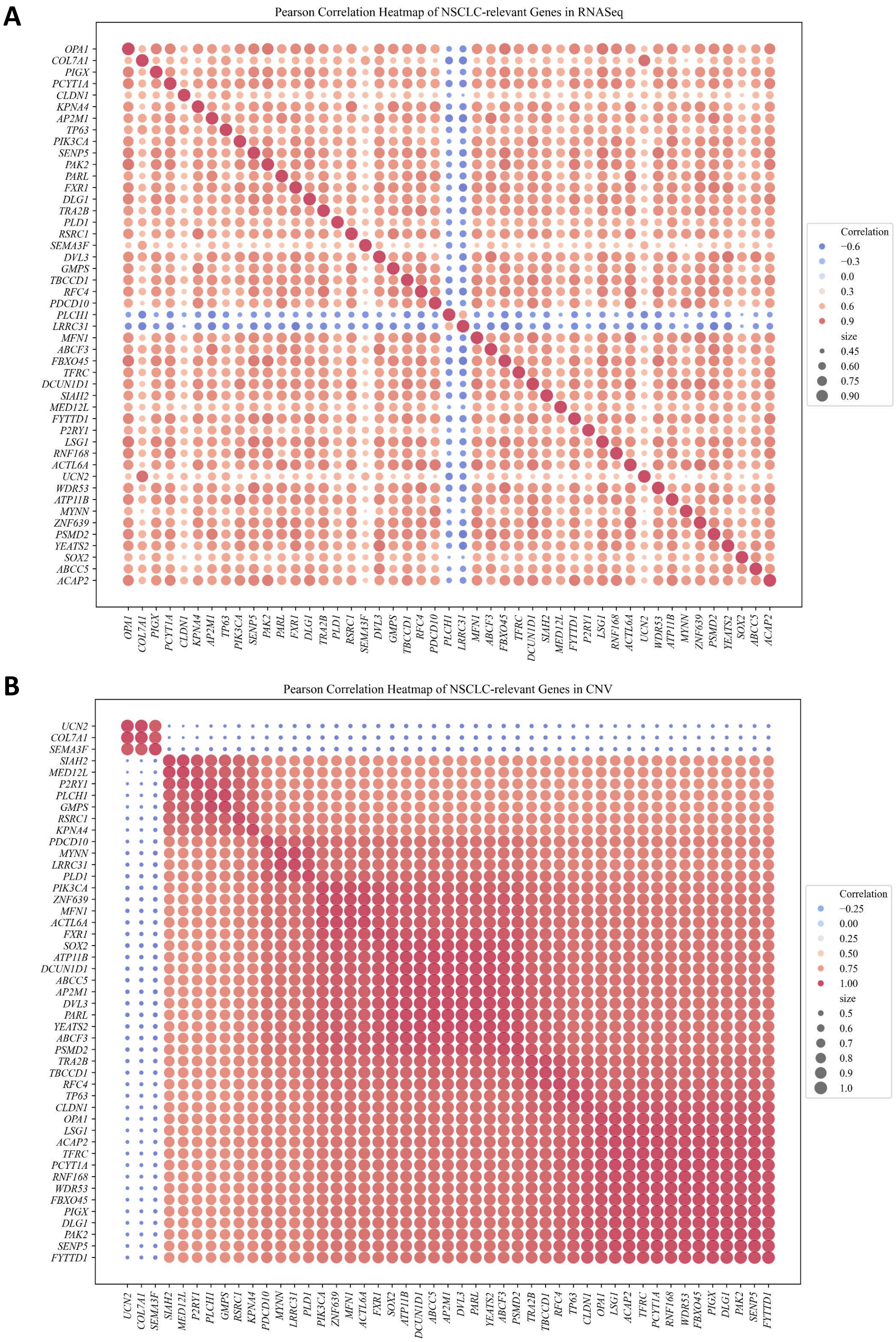

Supplementary Figure 2. Heatmap illustrating Pearson correlation between each identified gene in TCGA (A) RNASeq transcriptomics and (B) copy number variation (CNV) genomics data sets. Notably, the genes were found to be more positively correlated in genomics data compared to transcriptomics data. Exceptionally, *PLCH1* and *LRRC31* were found negatively correlated with the rest of the genes in transcriptomics data. Similarly, *UCN2, COL7A1* and *SEMA3F* were found to be negatively correlated with the rest of the genes in genomics data.

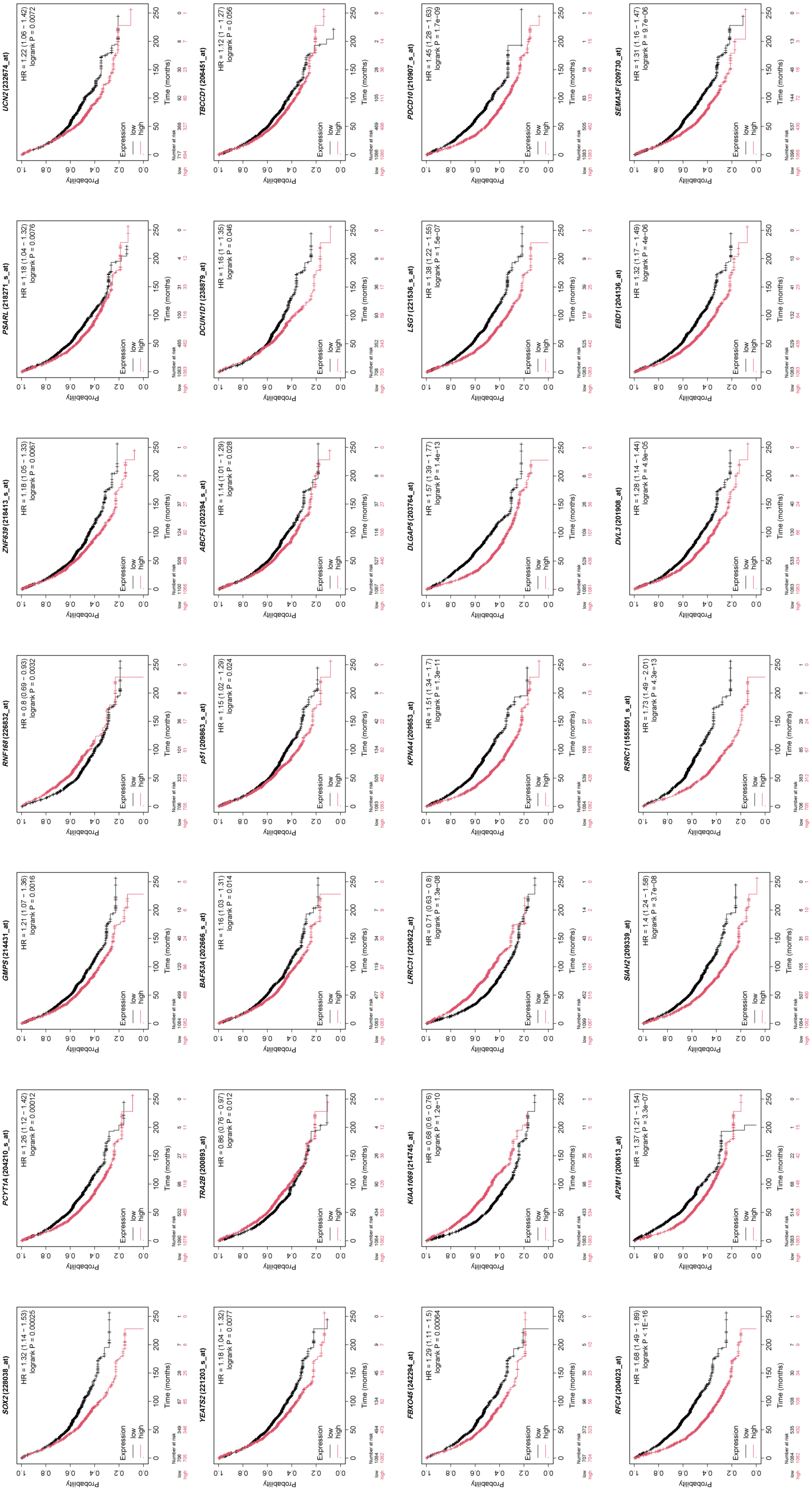

Supplementary Figure 3. Kaplan–Meier (KM) survival curves for 28 of the 47 identified NSCLC-relevant genes showing statistically significant associations with overall survival ($p \leq 0.05$). Patients were stratified into high- and low- gene expression groups. The red curves denote the high-expression group, while the black curves denote the low-expression group.
